# Bounded Justice and the Limits of Health Equity

**DOI:** 10.1017/jme.2021.34

**Published:** 2021

**Authors:** Melissa S. Creary

**Keywords:** Health Equity, Justice, Race, Brazil, Bioethics

## Abstract

Programs, policies, and technologies — particularly those concerned with health equity — are often designed with justice envisioned as the end goal. These policies or interventions, however, frequently fail to recognize how the beneficiaries have historically embodied the cumulative effects of marginalization, which undermines the effectiveness of the intended justice. These well-meaning attempts at justice are bounded by greater socio-historical constraints. Bounded justice suggests that it is impossible to attend to fairness, entitlement, and equity when the basic social and physical infrastructures underlying them have been eroded by racism and other historically entrenched isms. Using the case of Brazil’s National Health Policy for the Black Population, this paper proposes that bounded justice can contribute to justice discourses by serving as a concept, a proffering to a multi-disciplinary conceptual framework, and a potential analytic for those interested in the design of policy, technology, and programmatic interventions towards health equity.

## Introduction

Programs, policies, and technologies — particularly those concerned with health equity — are often designed with justice envisioned as the end goal. In fact, in the context of health, some practitioners hold health equity as the guiding force. According to public health scholars Braveman and Gruskin,^1^ “equity means social justice or fairness; it is an ethical concept, grounded in principles of distributive justice. Equity in health can be — and has widely been — defined as the absence of socially unjust or unfair health disparities.” As also intimated by Braveman,^2^ equity is linked with notions of fairness and ethical concepts of justice — particularly distributive justice. For purposes of health, distributive justice denotes a just distribution of resources according to the needs of the population. If health equity is measured by improving the health of the socially disadvantaged and their health is improved by a need-based distribution of resources, it is easy to think that all we need to address equity is a simple distribution of goods, materials, and resources. For justice-oriented policymakers and practitioners who want to ameliorate certain atrocities and indignities, particularly among marginalized populations, it makes logical sense to create policies or interventions that are equally justice-oriented. Literature shows us that these kinds of initiatives can and have been operationalized in many ways.^3^ These policies or interventions, however, frequently fail to recognize how the beneficiaries have historically embodied the cumulative effects of marginalization, which undermines the effectiveness of the intended justice. These well-meaning attempts at justice are bounded by greater socio-historical constraints. Bounded justice, then, suggests that it is impossible to attend to fairness, entitlement, and equity when the basic social and physical infrastructures underlying them have been eroded by racism and other historically entrenched -isms.

This paper proposes that bounded justice can contribute to justice discourses by serving as a concept, a proffering to a multi-disciplinary conceptual framework, and a potential analytic for those interested in the design of policy, technology, and programmatic interventions towards health equity. Brazilian health reform of the late 1980s and 1990s, which recognized that health was a right for everyone and paid specific attention to building health equity for disadvantaged Brazilians, effectively illuminates the mechanisms of bounded justice. The reconfiguration of responsibility for Brazil’s health was hard won by the “sanitary movement” — a group of health professional advocates embedded in the government and experts from academia determined to imbue equity into the public health system. While some scholars have argued that the constitutional mandate for universal access to health services and a bottom-up policy approach has actually led to inequity,^4^ I suggest that although some form of superficial justice may have been met, its reach has been limited, particularly for the intended audience. That is to say, though historic policy development processes were created to benefit the most marginalized, as we’ll see below, these processes have not been enough to counter the long-term effects of marginalization. As a result, many Afro-Brazilians, particularly those living with sickle cell disease (SCD), are glad to receive the relatively small (yet realistically quite meaningful) benefits that the Brazilian health policy distributes. Their response is not incommensurate with their awareness that these benefits are microscopic in comparison to the larger scope of civil rights that ought to be afforded to them as Afro-Brazilians. Studying the mechanisms of Brazil’s policy development and the contemporary implications for those living with SCD not only provides us with a case study of bounded justice as a concept and conceptual framework, but also sheds new light on the phenomenon of policy development itself.

Bounded justice is a concept that reveals the political idealism of equity-based policies by highlighting the realities of social, cultural, and economic norms. It calls for a closer investigation of how interpersonal processes of racism as a belief system and discrimination as an act have affected structural justice delivery. With its deployment comes a deeper understanding of the relationship between vertical equity and distributive justice, for instance. Bounded justice describes the inherent limitations of even forward-thinking and justice-based notions of inclusions. It is a concept and analytic that reveals how inclusive programs and policies often fail to recognize the fundamental, even existential, exclusion their target populations experience. This paper provides the biography of the concept grounded in the case of Brazil, its relationship to other concepts in an interpretative approach to social reality^5^ and a way to envision bounded justice as applied to the current moment of COVID-19 and the increased calls for equity as a solution to fix the ails of racial injustice.

## Bounded Justice as Concept

While the work of this paper is not to judge bounded justice as a “good concept,” following the rubric outlined by Gerring,^6^ bounded justice does meet the criteria of familiarity, resonance, coherence, differentiation, depth, theoretical utility, and field utility. Further, by Jabareen’s account,^7^ every concept has a history, contains components originating from other concepts, and relate back to other concepts. Bounded justice does indeed have a history; the chronology of its origins, for the purposes of this paper, occur alongside the re-design of the Brazilian constitution in 1988, though it is safe to assume that bounded justice has enacted itself in many instances, in differing socio-historic contexts, and temporally before the re-democratization of Brazil. Furthermore, this concept is connected to and problematizes well-developed concepts of justice, including aspects of distributive, restorative, and social justice, which will be discussed below.

In 2009, the Brazilian state implemented the National Health Policy for the Black Population (NHPBP) to “establish equity as a necessary pre-condition for the fulfillment of equality.”^8^ The text of the policy begins with guiding principles:

This policy is grounded in constitutional principles of citizenship and human dignity, the rejection of racism, and equality … With these principles are the joining of popular participation and social control, key tools for the formulation, implementation, evaluation and possible redirection of public health policies. These are developments of the principle of “community participation” and main object of Law 8,142 of December 28, 1990.

As 2020 drew to a close during the ongoing fallout of a pandemic that has exacerbated deep structural inequities merged with a national reckoning of racial injustice, the calls for justice (often intertwined with narratives of diversity, equity, and inclusion) have become more frequent and urgent.

This text links the contemporary policy to tenets written throughout the relatively new constitution of 1988: citizenship, rejection of racism, equality, all reinforced by the notion of “community participation.” The new constitution and public health system were created, in part, to address the country’s deep-rooted inequalities.

As the government moved towards democratization after a period of dictatorship, the opening of the political system encouraged more community engagement in health policy processes. This new openness allowed for strategically placed justice-oriented physicians *(sanataristas)* to campaign ardently for decentralization within the health sector of government. They integrated principles of “participation,” “inclusion,” and “equity” that served as the backdrop to bottom-up policy development and the creation of the *Sistema Único de Saúde,* Brazil’s Unified Health System.^9^ The new constitution called for public health services that consisted of an integrated, regionalized, and hierarchical network and constituted a single system organized by decentralization, comprehensive care with a preventive focus, and community participation.^10^ The role of the community — that is, individuals who lived with specific health conditions or advocates who represented these individuals — became more formalized in 1990, when legislation established national health councils and conferences at the three levels of government. In Brazil, this intentional pairing of the community, represented usually by civil society, with the elite processes of the government is known as *controle social* (social control). The legislation mandated that members of the community be directly involved with the creation, implementation, and evaluation of public policies. The *controle social* model came out of the work of two groups: socially mobilized citizens who were distrustful of an authoritarian and inaccessible regime, and a group of more elite health reformers, bent on addressing Brazil›s inequities. This community participation, also interpreted as an action towards inclusion, was justice driven by its *sanitarista* origin.

In short, the NHPBP was guided by the mission of a racialized health movement and drafted by individuals who demanded action from the state given the failures of equity production that the new constitution promised. The newly reformed health system prioritized social rights and called for citizen co-management of the system at the municipal, state, and federal levels. Yet, despite this mandate for citizen participation as a way to build in equity, the justice-based health policy was unable to address the underlying and deeper social inequalities embedded in individuals and communities, specifically those disadvantaged by racism, because attempts at health justice and reparation were bounded by larger societal, systemic, and structural factors that were not recognized as part of policy and system-building. Furthermore, Brazil’s constitutionally mandated justice-based health policies do not address the underlying mechanisms that generated initial historical inequalities. In Brazil, and around the world, the populations for whom justice is intended have lived with long-term marginalization and its cumulative impact. Bounded justice as a concept, therefore, reveals an important limitation: the policies, programs, interventions, and technologies that, at best, have historical reference points only go back so far, or, at worst, are completely ahistorical.

In essence, as Brazil’s reforms illustrate, and as the concept of bounded justice aims to show, attempts at health justice and reparation are limited in how effective their reach is. Health outcomes are the products and expressions of unjust social, economic, and political institutions. If health equity is social justice in health, as Braveman describes,^11^ it cannot be an isolated project meant to only remedy health-specific ails. It must be pursued in parallel with the attainment of justice in other and all spheres.^12^ Bounded justice challenges the idea that health equity is an achievable goal, given all of the other structural inequalities. The concept demands that policies and programs be designed with those constraints understood.

### A Seat at the Justice Table

As 2020 drew to a close during the ongoing fallout of a pandemic that has exacerbated deep structural inequities merged with a national reckoning of racial injustice, the calls for justice (often intertwined with narratives of diversity, equity, and inclusion) have become more frequent and urgent. In a special issue of *Science, Technology, & Human Values* on Entanglements of Science, Ethics, and Justice, Mamo and Fishman^13^ describe the tensions and complexities of justice and ethics, specifically as imbricated in techno-scientific practices and implicating practices of public health. Writing in 2013, the authors note, “‘justice’ frameworks continue to proliferate within nonprofit, philanthropic, and other organizations’ missions, often in concert with universities. Environmental justice, social justice, health justice, reproductive justice, sustainable justice, and other terms have emerged as focal points for twenty-first-century policy work and social activism.”^14^ Bounded justice as a concept necessarily critiques not only these tangential and emerging conceptions of justice but may aid in problematizing the established justice discourse as well. What follows is brief engagement with philosophies of justice in and out of relation with health, the ways they may intersect with equity processes, and how the concept of bounded justice helps us understand their limitations.

Rawls’ theory of justice as fairness describes a society of free citizens holding equal basic rights and cooperating within an egalitarian economic system — it is about how people are treated and how decisions are made.^15^ According to Labonté,^16^ “The first principle [of Rawls’ theory] is the ‘priority of the equal’ (basic liberties), which roughly equates with individuals having civil and political rights that protect them against excess authoritarian rule by the state. The second principle is based on legal equality of opportunity, which roughly equates with individuals having economic, cultural and social rights that obligate states to provide certain goods or services (the minimum resources or capabilities required to exercise one’s functionings).” However, as Daniels explains,^17^ “Rawls simplified the development of his theory [of justice] by postulating that all people function normally over a normal life span, barring the prospect of disease and disability from his social contract.” In *Just Health*, Daniels^18^ offers his own theory of justice and health based on three questions of justice: 1) What is the special moral importance of healthcare? 2) Which health inequalities are unjust? 3) When are limits to healthcare fair. It is in the answer to the second question that Daniels suggests that the promotion of social justice will aid in the establishment of equal liberties, equal opportunities, and fair distribution of resources (basics of Rawlsian justice). While Rawls’ theory of justice as fairness does not address health specifically, justice is central to the mission of public health — through health improvement for the population and fair treatment of the disadvantaged.^19^ An underlying value and principle of health equity is that health differences adversely affecting socially disadvantaged groups are particularly unacceptable because ill health can be an obstacle to overcoming social disadvantage — which suggests that need should be a key determinant of resource allocation for health.^20^ Many readers will be familiar with a graphic meant to illustrate the difference between equality and equity where three individuals of varying heights stand in front of a fence on either same-sized crates, which means that only the tallest of the three can see over the fence (depicting equality), or on stacked crates, so that they can each look over fence at the same height (depicting equity). The accompanying caption often reads something like “equity is a process and equality is an outcome of that process.” We, the interpreter, are to liken the crates to some type of resource: money, personnel, better access, better opportunity, policy — and the more crates, the more, we are to assume, resources. As a visual reminder of this distribution of resources, the individual is elevated by standing on the increasing crates. This is a representation of vertical equity, an attempt to achieve an equal outcome and in which “people are treated differently according to their initial endowments, resources, privileges or rights.”^21^

The concept of bounded justice critiques the vertical processes of distributing justice (and equity) and suggests that they do not go deep enough. Distributive justice is a tiered process that assumes that the intended recipient is able to adequately and efficiently accept and properly retain the benefits of justice. Equity in health is an ethical value, normative in theory, but often fails to structure practice. It is an ideal, and a policy goal, but the justice that practitioners seek is often imagined unrealistically. When programs are narrowly constructed to benefit marginalized populations without redressing the cumulative effects of disadvantage, the results constitute a bounded justice.

Anthropologist Anna Pagano^22^ has applied the term “compensatory justice” to describe the development of the NHPBP, but this term does not tell the whole story. According to Taylor,^23^ “The principle of compensatory justice is that, in order to restore the balance of justice when an injustice has been committed to a group of persons, some form of compensation or reparation must be made to that group.” This type of justice is restorative and attempts to restore the victim to the condition he was in before the unjust activity occurred. Brazil’s 2009 health policy, designed to redress the neglect often associated with health and Black populations,^24^ could be interpreted as health reparations designed to restore the Black population. But how does one restore a population deserving of reparation policy when the legacies of slavery caused the neglect? Those living with SCD in Brazil have argued that there would be no instance of disease were it not for the enslavement of African people (one of several sites where the disease is scientifically described to originate)^25^ brought to Brazil.^26^ The limited experience of some Afro-Brazilians in wielding political power is consequent of both personally mediated and institutional racism,^27^ as well as the legacy of the collective and cultural trauma of slavery.^28^ In Brazil, the provisions for justice set forth by the constitution, and contemporarily distributed, were limited by larger systemic forces. While the policy produced political optimism and excellent optics for the government, once implemented, it reified biological notions of race, was chronically underfunded, and failed to substantively engage with community stakeholders. The policy constitutes a form of bounded justice, a limited response sufficient to quiet critics but inadequate to reckon with historically entrenched realities.

Much can be found in contemporary literature around the race-based social policies that produced affirmative action directives for education in Brazil.^29^ These policies laid the groundwork for health regulations from the state in an attempt to address inequalities. Both the policy development practices of the reimagined constitution as well as the NHPBP demonstrate the bounded nature of justice in their associated distributive practices. “Affirmative redistribution” describes affirmative action reforms that tend to “seek to redress maldistribution by altering end-state patterns of allocation, without disturbing the underlying mechanisms that generate them.”^30^ In this context, the Brazilian state has attempted to provide equality of opportunity for health — first for all, but with an emphasis on the disadvantaged, and then specifically for vulnerable populations. For this opportunity to be just, vertical equity is required, meaning that “a disproportionate provision of public goods and capability resources for those whom history’s conquests, and today’s political institutions, place in highly unequal initial conditions” is necessary.^31^ But if social factors play a large role in determining our health, the resources needed to be healthy (the determinants of health) must be fairly distributed.^32^ In essence, the health policies designed for the marginalized populations in Brazil do little to address the underlying and upstream causes of poor health. Guimarães ^33^ affirms this lack of comprehensive attention as a trend by noting that while the Brazilian state made great efforts during the 1990s — initiated by President Cardoso’s promotion of racialized policies^34^ — it neglected to face the systematic racial inequalities demonstrated by income distribution and access to public services. Offering band-aid policies as metaphorical crates to elevate a population based on health-specific needs does indeed offer some level of justice, but if the elevation does nothing for the population’s equal or greater needs for access to formalized employment, safety in their communities, or better opportunities for education, the justice is limited.

In another interpretation of justice, restorative justice focuses on the harmful transgressions of perpetrators and actively involves victims and offenders in the process of reparation.^35^ In the case of the NHPBP, the offender is the state, on trial not only for its contemporary societal transgressions but also for the historical ones that trace back to the legacy of slavery. Further, while a restorative-justice-based reconciliation process is not taking place as it did in the intensive form of post-Apartheid South Africa, for example, the “victims” (patient constituencies) and “offenders” (administrators of the state via the public health apparatus) are working together as per the mandate of the constitution. In theory, genetic analyses deployed for reconciliation projects can nurture the group’s self-empowerment and “contribute to community cohesion, collective memory, or social transformation.”^36^ In reality, what we observe are ineffective practices of inclusion by the government — an offer for a seat at the table, accompanied with unstable and rotting chairs and no regard to how community participants may have arrived to the table, whether or not there is shared language to communicate across the table, and no accommodations for those whom may find the chair more harmful than helpful.

## Bounded Justice as a Conceptual Framework

Using Jabareen’s^37^ definition of a conceptual framework — a network of interlinked concepts that together provide a comprehensive understanding of a phenomenon or phenomena — I offer bounded justice. Bounded justice is inherently linked to a number of concepts that address the entanglement of justice, inclusion, and citizenship for vulnerable populations. Conceptual frameworks are not merely collections of concepts but, rather, constructs in which each concept plays an integral role.^38^ The concepts I envision that contribute to a bounded justice framework below all have slippages and tensions between them — there is a presumed relationship between them that lends itself to “an interpretative approach to social reality.”^39^

As such, bounded justice as a concept is created in concert with both well-established and novel schemas such as structural violence, ecological frameworks of health, intersectionality, embodiment, and biocultural citizenship. Each of these concepts offers distinct ways to help conceptualize how deployed justice tactics fall short while also working together to explain larger societal phenomena. The list of included concepts is not exhaustive, but the ones highlighted here each further contribute to the ways we value (or don’t value) lived experience in the explanation of health outcomes, policy development, and interventional design.

Structural violence is a concept introduced by Galtung^40^ and taken up by a number of scholars, most notably Farmer,^41^ who has used this construct to describe how manifestations of violence and harm can take place invisibly and systematically, rendering physical and emotional harm to groups of vulnerable populations. According to Whittle et al.,^42^ “integral to structural violence is the role of institutions and social practices in preventing such persons from reaching their full potential, emphasizing the capacity of the modern state to protect — or fail to protect — its citizens from large-scale forces of political economy and history.” One cannot take into account the modes of structural violence without considering the intersecting effects of colonialism, racism, patriarchy, and homophobia. Structural violence provides explanations for the ways that justice is bounded by “large-scale forces” and calls for deep excavation into the strategies that are developed for populations that have embodied these forces. Biocultural citizenship^43^ is useful here as well as a way to think about how racialized citizens strategically use their biological status and place-based cultural ties to hold the state culpable for their health status with full knowledge that the inequitable social milieu in which they are situated will never live up to their alleged status of belonging as ascribed by their birthright.

It is useful to think of biocultural citizenship as a continuum. This concept is a flexible mode of enacting belonging that varies depending on disease status, social class, and other identifiers and is enacted across the phenotypic continuum. So, too, is bounded justice. Too complex to situate as a polarity, bounded justice is a relational concept that necessarily draws from societal, cultural, and even biological factors — tailored specifically to product, place, and community. Structural violence increases or decreases at the hands of the state, and, in the context of Brazil’s policy development, there is an unstable attempt by the state to decrease the effects of historic structural violence. Petryna’s^44^ biological citizenship attends to how claims of suffering are bounded implicitly to actors seeking social equity. How might the collective trauma of slavery incite the government to respond with health policies as part of reparations to the traumatized? How can any policy appropriately attend to the suffering caused by this trauma? The life-course navigation through an inequitable society leads to biological consequences.^45^ Embodiment (particularly of social inequality) has long been considered an important concept in public health. Taking an eco-social approach to the ways in which we frame health has allowed many scholars to address health disparities (vs. inequities) in more tangible ways.^46^ Understanding how personal, situational, and sociocultural factors such as racism, sexism, classism, and homophobia intersect has far-reaching implications for policy development, knowledge production, and the design of interventions. These systems of oppression are mutually constituted and work together to produce inequality.^47^ The structural nature of violence manifests as unequal access to goods and services, differentiated access to income and educational potential, economic divestment, lack of political, cultural, and social capital, and systemic denial of civic and human rights.^48^ As Lane et al.^49^ write, “macro-level entities such as state and federal bureaucracies, health institutions, social environments, and social and health policies that form the context in which disproportionate illness and death occur” are often neglected when thinking about the distribution of resources in the name of equity. However, the historic, economic, political, and societal factors that have been recognized via these eco-social framings have led to scholarly outcries to focus health interventions and policies more towards systems and less towards individuals/populations.^50^ Even still, when a systems-approach policy designed to address social determinants of health, such as Health in All Policies (HiAP), is deployed, House^51^ found that “HiAP had not really been focused on all policies, but rather mainly on community-based (as opposed to national) efforts to affect a limited set of health behaviors. These community interventions generally failed to address the factors and policies that are most consequential for health, including disparities in socioeconomic resources and power, as well as problems of discrimination and segregation.”

The current charge of diversity, equity, and inclusion (DEI) initiatives should cause some to pause with worry as many actors respond to performativity politics. The “I” in DEI has been a particular interest to researchers and practitioners, as many try to understand and mitigate the embedded hesitancy often paired with inclusion strategies.^52^ Inclusion is a political performance of the present, meant to remedy a diagnosis that is both historical and embodied. We often think about inclusion in a vacuum and not as a fraction of a larger picture. We cannot, then, think about inclusion without interrogating exclusion. The concept of social exclusion is useful for inclusion in the framework because it takes into account an understanding of social processes and their consequences. Some scholars have linked social exclusion to inadequate social participation, lack of social integration, and lack of power resulting in unequal resources, reduced capabilities, and fewer claims on human rights.^53^ Social exclusion defines disadvantage as an outcome of social processes instead of assigning it as a characteristic of any one group.^54^ Much like the interweaving of the concepts above, the exclusion embodied by persons as a result of racialized or gendered differences will have an economic and material component as well and, for the purposes of this article (though with far-reaching implications), be attached to health outcomes. German sociologist Martin Kronauer ^55^ argues that social exclusion is a product of people’s relationships with the labor market, consumption, institutions, social relationships, culture, and geographical space. In public health, we would recognize this as an ecological framework of health and the basis of epigenetics. If we are prompted to design more pathways of inclusion due to the indignities of society and as a form of justice, we are also forced to reckon with the ways in which embodied exclusion will affect the efficaciousness of these designs — and in doing so, be conscious of the ways in which the design must counteract the inherent boundedness historically and contemporarily present.

Returning to the case of Brazil, we see that despite the constitutional mandate for community participation (inclusion) in policy development and evaluation, historically marginalized citizens still lacked power and capital and therefore were not seen as legitimate participants. Using Stinchcombe’s^56^ framing of legitimacy as associated with the use of state power to secure the support of “other centers of power,” we might imagine how the insidiousness and subconscious values of white supremacy could affect the political processes of inclusion as enacted by governmental elites. As parsed by Goodstein and Velamuri,^57^ Stinchcombe “recognized that institutions, including the state, represent structures, ‘in which powerful people are committed to some value or interest.’” There are two sides working in conjunction to produce bounded justice. On one side is a powerful state that, outside of a legislative mandate to address inequality, is not interested in redistributing power; on the other are the descendants of a population who have had their legal, economic, and general human rights historically denied, but have contemporarily been given political power in the absence of other important pieces of power. The new Brazilian Constitution of 1988 was aspirationally drafted by individuals embedded in a health reform movement and hopeful for its potential to enact change on the ground level. The legacies of elitism and white supremacy within the public health apparatus were stronger than the justice-based origins of equity building by embedded activists. As noted above, the NHPBP was drafted by individuals — and guided by the mission of a movement — who demanded action from the state given the failures of equity production that the new constitution promised. The anemic attempt at justice by the state, whereby bringing vulnerable populations to the table where legitimacy is unattainable, contributes to how equity processes, even well-intentioned ones, are bounded. Bounded justice as a conceptual framework reveals the political idealism of equity-based policies by highlighting the realities of social, cultural, and economic norms that are rooted in a racist society.

Though bounded justice was initially developed through the analysis of empirical data collected in Brazil, as a person who has worked with the sickle cell community in the role of a scientist, policymaker, and public health researcher for over 20 years, it is easy for me to see both the US-based and global implications of this framework. Bounded justice joins the above-mentioned concepts to form a conceptual framework with the understanding that the social and cultural elements of life are historically intertwined with the systems of power and inequality. It recognizes that health-related (and many other) programs, technologies, and policies are produced in these fraught political and economic fabrics with an intention to achieve justice, reconciliation, and reparation for vulnerable populations. Further, these productions are based on good intentions and usually as a countermeasure to the indignities and inequalities taking place among certain populations. Yet, these programs, technologies, and policies, which aim to bring vulnerable individuals to the proverbial table to have a better stake at their own health status, do not often take into consideration that the table is unwelcoming, and is not equipped to deal with, understand, or hear the individual’s total lived experiences that brought them to the table to begin with. The enthusiasm for representation disregards how social inequality in its broader contexts are brought to the table. Instead of taking into account just the pressures for health-related “diversity” in DEI initiatives — emphasizing alleged intentionality towards elevating the voices of the underserved in the development (or afterthought) of a program, policy, or technology — that energy must be turned to trying to understand how the broader social milieu of inequality has been inscribed in the very bodies being asked to participate. Practitioners and policymakers should know that the justice they are trying to introduce will always be bounded, limited by the social confines in which they are produced.

## Bounded Justice as a Diagnostic: Two Contemporary Cases

I have posed bounded justice as a concept and conceptual framework, both of which can have “methodological assumptions that can tell us about the ‘real’ world.”^58^ How could bounded justice be used as a potential analytical tool? Both the optics and reality of social justice in health (equity) are appealing. Those not concerned beyond the performativity of justice want to appear to have made at least superficial efforts, and those with actual investment are propelled — much like the *sanitaristas* of Brazil — by the call to address both historical and current inequities. Both can potentially do more damage than intended; neither will be able to completely deliver the justice they claim. There is a need for a diagnostic and eventual praxis — a systematic process involving the continual interplay between self-reflection and action — to help those who want to endeavor into practices of justice or for those who want to evaluate the actions of others who promise the delivery of justice.

As academic institutions take on an urgent commitment to equity and inclusion, and as hospital systems and communities begin to undertake efforts to reconstruct how communities were and are disparately impacted by the pandemic, efforts to include marginalized communities in this reckoning are susceptible to the same dynamics that exacerbated the pandemic’s impacts on these communities in the first place.

If we were to treat injustice metaphorically as a disease, we would be served well by Rosenberg’s^59^ analysis of the framing of disease. The diagnosis of disease organizes therapeutics and legitimates the existence of the disease as well as any subsequent interventional public policy. Diagnosis provides clues from the past and guides the physician’s treatment and the patient’s expectations around entitlements and reimbursements. According to Rosenberg,^60^ “From the patient’s perspective, diagnostic events are never static. They always imply consequences for the future and often reflect upon the past. They constitute a structuring element in an ongoing narrative, an individual’s particular trajectory of health or sickness, recovery or death.” Bounded justice as a diagnostic for injustice has implications for the appropriate and deliberate creation of programs, policies, and technology; can provide guidance for responsible and more effective inclusion; and can evaluate entitlement processes. What follows is a brief analysis of two contemporary cases where bounded justice is deployed as a conceptual framework and diagnostic. One case deploys the analytic of bounded justice for the recent demands for justice in response to the global pandemic of COVID-19; the other case analyzes the trajectory of “call to actions” for SCD in the United States context.

### COVID-19 and the Exposure of Scar Tissue

Bounded justice recognizes that the call to action to insert justice for the populations most affected by the COVID-19 crisis must account for the structural barriers that have been at play long before the threat of viral infection entered these communities. In spring 2020, the world experienced the onset of a global pandemic with the rapid spread of COVID-19. While it originated in Wuhan, China, this coronavirus quickly spread to the United States and rapidly exposed the already existing fault lines of inequity throughout our society, especially in our healthcare system. As the number of those infected began to rise across the country, the pattern of disproportionate burden revealed itself in Black and Brown bodies, and cities with large Black and nonwhite Hispanic populations emerged as hot spots for the spread of the virus. Individuals and institutions began to clamor for data attached to race. In late March 2020, Democratic lawmakers called for comprehensive demographic data on people who are tested or treated for the virus that causes COVID-19. These lawmakers, accompanied by medical practitioners, policymakers, and activists alike, argued that the lack of data was a hindrance to the monitoring, tracking, and action towards the elimination of health disparities. Race-based data can be helpful. It has the potential to tell us where resources — money, personnel, ventilators, messaging — should be directed. Shortly after the call for data, the White House Coronavirus Task Force declared that Black Americans are disproportionately impacted by the coronavirus pandemic due to “underlying medical conditions…the diabetes, the hypertension, the obesity, the asthma. Those are the kind of things that wind them up in the ICU and ultimately give the higher death rate.”^61^ What truly underlies this narrative are structural inequities.

The embodiment of social inequities, even outside the realm of health, is stark in the case of Baltimore, Maryland, in the aftermath of Freddie Gray’s death in 2015. Freddie Gray was a twenty-five-year-old Black man who died of a spinal cord injury while in custody of the police. The anger associated with city-wide protests and riots centered on the localized lack of answers surrounding his death coupled with the city’s troubled history with police brutality and was set within a larger conversation on the nationally systematic patterns of criminal injustice for Black men. As a response to this unrest, the Obama White House called for an advisory committee to address existing and deep racial disparities. Then-representative Elijah Cummings was quoted as saying, “How do we take this moment, put the spotlight on Baltimore, and try to make things right as fast as we could?”^62^ Millions of dollars to address violence, transportation, job attainment, and education (social determinants of health) were invested in the city. Despite the aid and attention from all levels of government, philanthropic groups, and private organizations, the equity-based initiative was crippled under the weight of generations of “deep distrust of institutions, unstable political leadership, and the intractable barriers erected by generations of poverty, segregation and disinvestment … There was just too much scar tissue.”^63^

This metaphorical “scar tissue” is indeed the obstacle to the justice delivery system or the way we envision the distribution of resources (the proverbial crates). The resources needed to address the ongoing and future crises of COVID-19 in the communities that will need it most will have to be distributed with bounded justice in mind. Using bounded justice as an analytic prepares experts for the fact that antibody testing or vaccine distribution, even if equitably allocated, will have to contend with larger societal concerns that consider local infrastructure, historicized labor practices, and collective trauma. It takes into account that the community taskforce, even when thoughtfully convened, when not given time to develop trust or identify common values, will hold back, be easily dismissed, or lose interest. Bounded for eventual divestment of resources once there was sustained linkage of COVID-19 to blackness.^64^ In the coming days, as task forces, advisory committees, strategic planning sessions, and academic interventionists convene with justice in mind for the communities in most need, the framework of bounded justice should serve as a reminder to consider the ways in which injustice, disadvantage, and marginalization are constant variables. How do we build effective policies and programs designing with these deficits in mind? How do we alter what we ask for or are prepared to give in order to adequately approach the deep-rooted nature of equity building?

As academic institutions take on an urgent commitment to equity and inclusion, and as hospital systems and communities begin to undertake efforts to reconstruct how communities were and are disparately impacted by the pandemic, efforts to include marginalized communities in this reckoning are susceptible to the same dynamics that exacerbated the pandemic’s impacts on these communities in the first place. Consider the following hypothetical case of 2021 vaccine distribution post–COVID-19 pandemic in Atlanta, Georgia. Metropolitan Atlanta area zip codes disproportionately shoulder the burden of COVID-19 cases. Throughout Cobb, Dekalb, Fulton, and Gwinnett counties, zip code burden is mapped along racial lines. Scientists and others have already called for equitable and just distribution, prioritizing high-risk populations who may have underlying complications and increased risk of disease transmission.^65^ Data suggest that these high-prioritized populations will be African American.^66^ As industry, public health officials and community members convene to develop policies, public health campaigns, and vaccine dissemination plans, an equity-based approach will have to take more than geospatiality into account. The justice will be limited unless stakeholders consider not only the general discourse of public confidence in vaccines, but its specific applications to the African American and African diasporic population scattered throughout metropolitan areas. These include well-known factors such as suspicion and mistrust, but also an added regional tension between city and state political officials. Effective implementation requires transparent conversations that explicitly discuss racism and engage with long-term, trusted members of the community. An historical analysis of resource allocation by local legislators should take place to assess patterns of community investment. Budgets are moral documents, and financing must reach beyond the development of the vaccine to include subsidiaries and stipends to address lack of transportation and lack of working on these acute strategies to address vaccine hesitancy there must also be, in parallel, action taken to address the even more complex and deep-rooted chronic concerns of hyper surveillance, food insecurity, racial capitalism, and segregation. All efforts will require substantial amounts of time and material resources, means of sustainability, and commitment to community building.

### Sickle Cell Disease and the Constant Quest for Equity

In the medical literature, sickle cell disease (SCD) and cystic fibrosis (CF) are often compared for both their similarity in genetic inheritance as well as difference in prevalence demographics. In two 1970 editorials published in the *Journal of the American Medical Association* and the *New England Journal of Medicine*, Roland Scott, a leading African American physician, highlighted a substantial difference in the research effort for sickle cell anemia compared with other chronic childhood diseases.^67^ He noted that, in 1967, volunteer organizations had raised $1.9 million for CF research, a primarily white disease that only strikes 1 in every 2,940 people, while less than $50,000 was raised for SCD, a disease that affected as many as 1 in 400 people at the time. In his articles, he emphasized the relationship between the status of SCD research and civil rights and suggested that disparities in research funding for this predominantly Black disease was an issue of social justice. Despite the passing of the National Sickle Cell Anemia Control Act in 1971 and several other legislative initiatives passed since then, the disparity between federal and private funding persists. In the 2020 *Strategic Plan and Blueprint for Action*,^68^ a *National Academies of Sciences, Engineering, and Medicine* proposal to address SCD, CF was recognized again for being rare and having well-organized and well-funded healthcare delivery systems despite having less prevalence. SCD is a biologically based disease that is not race limited, yet is associated with differential investment and treatment, which likely reveals racism as a root cause for disparity.

Racism is indeed named throughout the 522-page report and recognized as a contributor to the challenge of stifled progress towards funding. Racism influenced the committee’s decision to add that “health care be ethical” as a foundational principle to their vision for people living with SCD.^69^ Racism was named as the cause of implicit bias and stigmatization within and outside the healthcare system, magnifying the burden caused by low socioeconomic status found throughout the population living with SCD. Dedicating a whole section to racism, the report states, “Because SCD is found mostly among black individuals globally, it is inevitably linked to racism and health inequity.”^70^ Equity is also a foundational principle undergirding the committee’s scope in addressing SCD and its challenges. Despite this charge, if we are reminded that health equity is social justice in health, we must be reminded, too, of the ways justice is limited even in the thoroughness of the strategies outlined for SCD in this report. Bounded justice provides the tension between the acknowledgement of racism’s impact on the advancement of SCD and simultaneous recommendation of the Cystic Fibrosis Foundation to serve as a model for the community. Without understanding the ways in which social, political, and economic capital are embedded in the primarily white bodies involved in the narrative of CF, this tension can never be addressed. The private donations, pharmaceutical and other investments made into the scientific and philanthropic ecosystem for CF involves stakeholders, researchers, providers, and patients who lack the legacies of deprivation and exclusion. Further, even though the report provides twenty-four recommendations that span themes such as more education for educators, better incentives for potential physicians, enhanced vocational training opportunities, and data collection development, there is no mention of how, in parallel, we must also actively attend to racial bias and discrimination in the school and healthcare systems, and acknowledge the societal factors external to poor health that lead to missed educational opportunities. Sociologist Ruha Benjamin^71^ asks, “How, if at all, do people’s experiences of everyday policing relate to their trust of other social institutions?” Benjamin asks this in the context of her study on patients living with SCD and their interactions with the clinical research and medical system. How can we expect a robust patient-centered data registry in which individuals are hyper-valued for their data but undervalued otherwise within the healthcare system and society more broadly?

SCD has long struggled for recognition and advancement; challenges have been repeatedly called out and then paired with claims towards amelioration as if the initial recognition of challenge never occurred in the first place. The Academies report is important and lays out a “blueprint for action,” but there have been many “blueprints,” “calls to action,” and “priority agendas” laid out for SCD many times over. A Sickle Cell Summit meeting was held in 2007, “inspired by the recognition of healthcare disparity — both in the clinical care of persons with sickle cell disease (SCD) and in the research efforts directed toward understanding and treating this condition.”^72^ Sponsored by the American Society of Pediatric Hematology Oncology, the resulting position paper laid out similar recommendations that covered adequate and appropriate healthcare professionals, collaboration between federal agencies, and enhanced role of the community. While this report was not as comprehensive as the Academies report, the point is that efforts are often duplicated as a result of short memories, little action, and ineffective means of sustainability. This, too, is a form of bounded justice.

In September 2020, during Sickle Cell Awareness Month, Admiral Brett Giroir, Assistant Secretary for Health and newest White House champion, wrote about the state of SCD care in the United States: “Early in 2018, we established an interagency Health and Human Service (HHS) SCD work group to coordinate activities across existing federal programs, reduce duplication, and develop bold, transformative initiatives.”^73^ In September 2011, then-Secretary of Health Kathleen Sibelius made SCD a HHS an institutional priority, promising to create a comprehensive database, improve the care of individuals with SCD, and better engage national and community-based advocacy efforts. In fact, the then-Directors of the National Heart Lung and Blood Institute at the National Institutes of Health (NIH) also wrote an editorial in 2012 titled “Future Directions of Sickle Cell Disease Research: The NIH Perspective”^74^ in which they lauded HHS’ efforts. This loop of inaction is bedfellows with public performativity — a written or visual display of commitment to the same thing over and over again (for SCD during the national month of recognition), all the while never moving the proverbial needle enough to make a difference. Further, there is no recognition of the historical efforts that have taken place. As a result, those new to the loop of inaction are promised the same things that have already promised, but with a fresh set of stakeholders who are enraged by the inaction and with it fresh attention for the performance of shared institutional disbelief and alleged commitment — all the while conveniently forgetting that the same exact processes have occurred many times over before an administration, leadership board, or health official decides it’s the cause du jour again. In 2006, Lauren Smith,^75^ a pediatrician, penned an article making the comparison again between SDC and CF — serving as a midpoint between Roland Scott’s original inquiry of inequity and the Academies’ repeat observation. She and colleagues questioned the equity of funding as well as the equity of quality of care. The same things that Dr. Scott recognized in 1970 are still major challenges fifty years later. This continued denial of equity for SCD in the United States may take a different form than the denial of equity in Brazil, but both are fueled by racism. Camara Jones,^76^ renowned scholar on racism and health, states, “institutionalized racism is often evident as inaction in the face of need.” We see clearly, then, in the context of the US how this might play out. In the case of Brazil, it is, in fact, an acknowledgement of institutional racism that leads to mandated equity building, but its efforts are eroded by the stronger forces of racism. In both cases, social justice in health is thwarted.

## Conclusion

The concept of bounded justice helps conceptualize the limits of the distribution of health-based rights when they are impinged upon by societal forces. This by no means is a suggestion that public health practitioners, policymakers, and activists curb their efforts in fighting for equity. Social justice is the foundation of public health, and the quest for equity must not cease. However, currently there is little attention paid to the ethical calculus of equity attempts in health. While much literature claims a direct link between justice and health equity, there is little written interrogation about the actual efficacy of the presumed delivered justice. We don’t have good interventions that help us determine if the intended justice has been met. The concept of bounded justice is useful because it provides a way of theorizing how embodied outcomes of accumulated injustice and exclusion inhibit the receipt of justice even via intentional, well-meaning, well-researched programs, policies, and technologies. It thereby allows us to better consider the realities of the intended benefitting constituents. The goal of this paper was not to lay out examples of structural barriers. Public health has long been aware of the systemic and institutionalized opponents of good health outcomes. Policy interventions such as HiAP are evidence of this knowledge — though even these are bounded. The intention of this paper is to repudiate empty efforts of inclusion and to complicate the aspirations of social justice.

Bounded justice is embedded in a larger conversation around the politics of need and the mobilization of moral arguments around suffering. This concept also has far-reaching implications for social policies outside of the realm of health as well (e.g., food policy, educational interventions, housing). Bounded justice has wide relevance as a praxis to help quantify justice, contribute to research and conceptual ethics, to serve as a lens from which to evaluate policies, and as a biopolitical lever to raise awareness of unforeseen consequences of our current policies and practices. There may be a sense of frustration at the lack of a bounded justice checklist. We must be mindful of how we are prescriptively trained to respond immediately to injustices as they become more apparent. The sense of urgency is a natural one, but one “that makes it difficult to take time to be inclusive, encourage democratic and/or thoughtful decision-making, to think long-term, to consider consequences.”^77^ In reality, there is no quick fix to address the deep rootedness of inadequate equity processes.

How do we adequately frame and fight for health equity as a human right? Even if equity in unattainable in completeness, as bounded justice suggests, it is our role as policymakers, practitioners, interventionists, and technologists, to design programs, policies, interventions, and technology with this limitation in mind, and then redesign as necessary. If praxis means“moving back and forth in a critical way between reflecting and acting on the world,” and we are compelled to create a more just world, we must theorize, study, evaluate, re-design, re-evaluate, act and then return to the cycle — with reflection embedded throughout — to determine the most effective ways to distribute justice.

Consider the *Framework for Equitable Allocation of COVID-19 Vaccine*,^78^ co-created by experts in epidemiology, community engagement, health equity, law, and bioethics. Distilled in a 273-page report, the Committee on Equitable Allocation of Vaccine for the Novel Coronavirus offered seven recommendations. Intentionally centering equity as a guide for the framework, it offers a rigorous manual to follow in effort to “to assist policy makers in the domestic and global health communities in planning for equitable allocation of vaccines against severe acute respiratory syndrome coronavirus 2 (SARS-CoV-2).”^79^ Despite the amount of labor and expertise it took it design this report, it was still created, one must assume, in a short amount of time given the timeline of COVID-19 events in the United States. Besides the urgency that fueled this report, we must also take into account the historical, political, and societal aspects of each place where the recommendations might be implemented. The justice entangled in the equity processes of this framework will still be bounded. The undertaking of “unbinding” justice might be akin to the development and implementation of impact assessments as discussed by Osagie Obasogie ^80^ in *Beyond Bioethics: Toward a New Biopolitics*. Impact assessments are evaluative mechanisms used by government agencies to analyze the risks and benefits of new proposals so as to promote individual and social well-being. Most notable are environmental impact assessment, but health, social, and human rights impact assessments have also been deployed by various governmental stakeholders. These assessments are evidence-based, multidisciplinary, and include multiple stakeholders. A justice impact assessment would also take on these characteristics, with an anti-racist framing to guide the processes. In his proposal for race impact assessments,^81^ asserts, “mature impact assessments require the collaboration of experts across multiple fields and affected stakeholders to create model tools that balance new technologies’ potential benefits with the potential risks of reifying social categories of race as biologically significant lines of human difference.” While these assessments are meant to assist policymakers on a governmental level, I envision a justice impact assessment to also be tailor-made for engineers who may want to design technologies for medically underserved, for funders who want to assess the final product of their awardee’s efforts, for activists who want to assess the work of those making promises for their communities, and for public health departments that design those community-level interventions for equity. Whomever the audience, what remains is deep entanglement with stakeholders that takes into account the long histories of systemic oppression. Like other impact assessment models, the process could be lengthy and take years. We cannot attend to equity purposefully with the ease of a checklist and urgency of a quick fix.

While I believe there is research to be done to help develop an evaluative tool that can assess how equitable or inclusive certain policies and interventions are being enacted, there is space for us to be mindful now of the vertical processes of distributing justice. There is also a call for new language for the “interventions” designed for justice in health (equity). An intervention is usually time limited and seeks to change a specific behavior or habit. Due to the deep-rooted societal forces bounded justice wants us to contend with, those of us invested in the work of unbinding justice must reframe our approach. What I am suggesting is that there is a relational positioning between vertical equity (justice distribution) and how deeply we must dig to unearth the real needs of a community. We cannot attend to policies specific to sickle cell disease or COVID-19 without first recognizing the ways that deprivation and social exclusion are embodied as a result of colonialism and slavery onto bodies who are also contending with a medical diagnosis. That is not to say that in the moment that we do not, in parallel, address the urgency of the acute ailment for populations so obviously in need, but that we are attentive to both the acute and chronic, the longer deeper “interventions” that true equity calls for. These problems will call for both biomedical and public health interventions that address emergent health crises and necessarily must be critiqued with a bounded justice lens. Simultaneously, we must attend to the much longer course of action of dismantling systemic oppressions by unlearning disciplinary norms, becoming anti-racist, changing laws and the ways that they are conceptualized, and creating sustainable and multigenerational relationships based on trust.

During my fieldwork in Brazil, I sometimes heard the frustrations of those in the Black Movement who felt that sickle cell disease had received its fair share of attention. While they understood the disease’s utility in serving as a clear representative for blackness, they also felt it was now time to focus on other issues related to Black health — most remarkably concerns around violent deaths. Members of the Black Movement also understood violence as a social determinant of health and a real concern for the Afro-Brazilian population. Violence, in addition to a cadre of other societal forces such as discrimination, lack of access to education, and consequently reduced income, have been continuously reported to disproportionately impact Blacks in Brazil. Bounded justice, then, also captures this essence of day-to-day injustices that occur within the Black Brazilian population that remain unaddressed even with the mandated distribution of health rights.

According to Krieger,^82^ key to addressing critical questions about the existence of injustice are the “conceptual frameworks people develop to use to understand, investigate, and alter the social and ecological worlds in which we live.” Bounded justice requires us to engage with critical thinking and critical reflexivity about the ways that nation-states, institutions and individuals rely on both the hope and aspiration of equity, as well as the ease of performativity around the calls for — equal attention to the deep socio-historical injustices, in parallel to the quicker fixes that a moment may call for — is daunting and overwhelming. How do we address colonialism as the root of inequity? How do we adequately frame and fight for health equity as a human right? Even if equity in unattainable in completeness, as bounded justice suggests, it is our role as policymakers, practitioners, interventionists, and technologists, to design programs, policies, interventions, and technology with this limitation in mind, and then redesign as necessary. If praxis means, “moving back and forth in a critical way between reflecting and acting on the world,”^83^ and we are compelled to create a more just world, we must theorize, study, evaluate, redesign, re-evaluate, act and then return to the cycle — with reflection embedded throughout — to determinthe most effective ways to distribute justice.

## Note

The author has received consulting fees and general honoraria associated with this manuscript.
